# Evaluation of the Impact and Fracture Toughness of a Nanostructured Bainitic Steel with Low Retained Austenite Content

**DOI:** 10.3390/ma16052003

**Published:** 2023-02-28

**Authors:** Mihael Brunčko, Peter Kirbiš, Ivan Anžel, Leo Gusel, Darja Feizpour, Tomaž Irgolič, Tomaž Vuherer

**Affiliations:** 1 Faculty of Mechanical Engineering, University of Maribor, Smetanova 17, 2000 Maribor, Slovenia; 2 Institute of Metals and Technology, Lepi pot 11, 1000 Ljubljana, Slovenia

**Keywords:** evaluation of fracture toughness, impact toughness, nanostructured bainite, natural aging, low retained austenite content, kinetically activated bainite KAB

## Abstract

The impact and fracture toughness of a nanostructured, kinetically activated bainitic steel was determined using Standard methods. Prior to testing, the steel was quenched in oil and aged naturally for a period of 10 days in order to obtain a fully bainitic microstructure with a retained austenite content below 1%, resulting in a high hardness of 62HRC. The high hardness originated from the very fine microstructure of bainitic ferrite plates formed at low temperatures. It was determined that the impact toughness of the steel in the fully aged condition improved remarkably, whereas the fracture toughness was in line with expectations based on the extrapolated data available in the literature. This suggests that a very fine microstructure is most beneficial to rapid loading conditions, whereas material flaws such as coarse nitrides and non-metallic inclusions are the major limitation for obtaining a high fracture toughness.

## 1. Introduction

It is known that low-alloy TRIP (transformation induced plasticity)-assisted steels have superior impact properties compared to ferrite and ferrite pearlite steels and can even approach those of maraging grades. Up to 80% of the fracture toughness, measured in terms of the crack extension force, of fully austenitic grades can be attributed to the TRIP effect [1]. These benefits are obtainable, providing the austenite has a sufficiently high stability to transform under the influence of deformation rather than stress [2,3].

The contribution of the TRIP effect in multiphase microstructures consisting of lower bainite and retained austenite, however, is not entirely clear, as the cracks are thought to propagate mainly along the interphase boundaries, behaving in a manner similar to dual-phase (DP) steels [4]. In this regard, the influence of the retained austenite’s stability is considered to be negligible [4]. In other studies, some improvements have, nevertheless, been reported, compared to similar steels in which transformation of retained austenite does not occur [2]. It is generally accepted that a carbide-free lower bainite microstructure exhibits superior fracture toughness compared to carbide-free upper bainite, analogous to conventional bainitic steels containing cementite. However, the reverse might be true if the carbide-free lower bainite structure contains relatively coarse blocks of retained austenite, as these can exhibit a stress-induced transformation into coarse high carbon martensite [5].

In the vast majority of engineering steels containing retained austenite, the TRIP effect can be explained in terms of stress-induced transformations [6]. Even though, arguably, some minor strain is required, there is no clear correlation between the deformation and the amount of retained austenite which is transformed. The mechanical component of the driving force is dependent on the stress state, and the highest possible values are given approximately by the following relations [7]:

(1) $$\Delta G_{\sigma }^{\gamma \alpha }=-0.86\sigma_{tension}\;\left[\frac{J}{\mathrm{mol}}\right]$$

(2) $$\Delta G_{\sigma }^{\gamma \alpha }=-0.58\sigma {\;}_{compression\;}\left[\frac{J}{\mathrm{mol}}\right]$$

(3) $$\Delta G_{\sigma }^{\gamma \alpha }=-1.42\sigma_{stress\;at\;crack\;tip}\;\left[\frac{J}{\mathrm{mol}}\right]\;$$

where the driving force $\Delta G_{\sigma }^{\gamma \alpha }$ is in J mol^−1^, and σ is the nominal elastic stress [MPa].

For any given stress state, temperature and strength level, there is a certain austenite stability that results in the optimal TRIP effect. Otherwise, the austenite may deform too much for the resulting martensite to provide sufficient hardening, and thereby transfer the stress to the remaining austenite phase, enabling it to deform and prevent the onset of necking [8].

In the context of ultra-high strength and nanostructured bainitic steels, there is an indication towards a beneficial effect of retained austenite, especially within grades transformed at higher transformation temperatures [9,10]. Nanostructured bainite refers to when the thickness of the bainitic-ferrite plates is in the order of a few tens of nm, or finer. When such steels are tempered to transform the retained austenite phase fully, a substantial decrease in fracture toughness is observed, despite the lower hardness in the tempered condition [11].

Taking the stability of retained austenite into account, it would seem that the scope of application for such steels is somewhat narrow. Additionally, there are very few data available in the literature exploring tensile behavior below room temperature. However, as we approach tensile strengths in excess of 2 GPa, too-high austenite stability is no longer commonly observed, and it becomes challenging to ensure sufficiently high stability without reducing the rate of bainite formation beyond acceptable limits. In fact, the ductility has often been shown to improve when the tensile tests were conducted at higher temperatures; for instance, at 200 °C [12]. This would suggest the stability of the retained austenite is often suboptimal at room temperature. It has been shown previously, using the example of nanostructured bainitic steels forming kinetically activated bainite (KAB), that the impact toughness increases dramatically when the retained austenite fraction is reduced to an amount below 10% [13], which corresponds to the critical value at which the retained austenite ceases to be a continuous phase. It is, however, counterintuitive that these two properties would be mutually exclusive, thus stimulating further research.

In light of these results, it is now of interest to determine how a very low retained austenite content would influence the steel’s fracture toughness. The aim of this work is to evaluate the fracture toughness of the newly developed nanostructured bainitic steel in relation to the retained austenite content and hardness.

## 2. Materials and Methods

**Figure 1:**
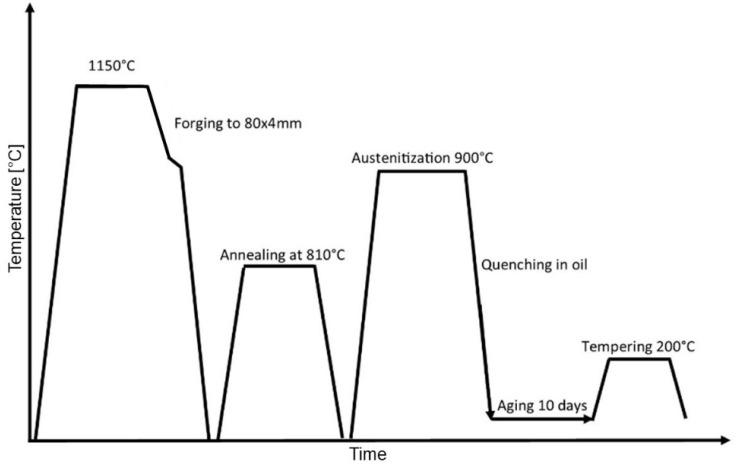
Heat treatment of steel.

The steel samples were produced in the form of 10 kg ingots, which were vacuum induction melted from commercially pure steels and alloying additions. In the current work, an alloy is considered with the following nominal chemical composition shown in Table 1:

The ingots were reheated to 1150 °C and forged to final dimensions of 80 × 4 mm. After forging, the billets were cooled in air to room temperature, followed by annealing and final heat treatment by reheating to 900 °C and quenching in oil. After quenching, the billets were aged at room temperature for a period of 10 days in order to reduce the retained austenite fraction to below 1%, followed by tempering at 200 °C as depicted in Figure 1, after which a final hardness of 62 HRC was obtained.

The billets were then machined to a thickness of 2.5 mm to remove the uneven surface and the decarburized layer, after which specimens were prepared for testing.

The impact toughness was evaluated using unnotched ¼ size specimens of dimensions 10 × 2.5 × 55 mm in acc. with ASTM E23. This type of sample was chosen in order to ensure the same material thickness was used throughout the whole analysis. The samples for the testing of tensile properties were prepared using EDM (electrical discharge machining) cutting and were ground to final dimensions acc. to ASTM A370 and tested in compliance with the Standard. The hardness values were measured using the Rockwell C method according to ASTM E 18-07.

The samples for light microscopy were ground using SiC paper to P2400, followed by polishing using 3 µm and 1 µm diamond suspensions, the final step being 0.05 µm alumina suspensions. For the FESEM (field emission scanning electron microscope) characterization, the metallographically prepared samples were etched with Vilella, gold sputtered and observed using a FEI Sirion NC 400, which was also used to observe the fracture surface.

The samples for high-resolution transmission electron microscopy (HRTEM) were cut into 3 mm disks of 1 mm thickness using a slow-speed saw, and ground carefully to about 0.5 mm, followed by additional thinning using the procedure of electro polishing and light etching, observed using a Jeol JEM-2100.

The phase fractions of ferrite and retained austenite were determined using X-ray diffraction on a Bruker D8 Advance, operating using a Cu anode at 40 kV/40 mA and a secondary graphite monochromator, within a 2 theta range between 40° and 90° at a scan rate of 0.5°/min. Prior to the measurements, the samples were deep-etched using a Villella reagent in order to remove any deformed surface layer.

The fracture toughness was obtained by testing using SENB (single edge notch bending) specimens in compliance with Standard ASTM E-399 [14], using samples of 2.5 mm thickness. A notch was EDM machined and the sample pre-cracked by bending on a dynamometer. After the initial crack had grown to the desired length, the crack was propagated by bending in a tensile testing machine. The sample was, afterwards, broken on the dynamometer and the crack surface observed using a Keyence VHX 5000 digital microscope, and the different regions observed carefully. The cracked surface was then evaluated by FESEM microscopy.

To determine the fracture toughness of the material, we followed the ASTM E1820a procedure, which is suitable for more ductile and tough materials. For brittle materials, the fracture toughness *K_IC_* is directly obtainable by using the ASTM E399 Standard, where the plane strain condition at the crack tip is checked by the relation *B*, *a*_0_, *b*_0_, ≥2.5 (*K*/*σ_Y_*)^2^. In the current work, fracture toughness *K_JIC_* was obtained from the J-integral using the single specimen method and the “Normalization Data Reduction Technique”, as described in the ASTM E1820a-Standard. When evaluating *K_JIC_* from the J integral, the conditions required by ASTM E1820a were fulfilled, such as pre-crack nucleation from a machined notch, crack length and shape of the initial crack *a*_0_, crack length and shape of the physical crack *a_p_*, geometrical characteristics *B* and *b*_0_, etc. When analyzing the specimen thickness *B* and initial ligament *b*_0_, both magnitudes satisfied the plane strain condition at the crack tip of the SENB specimen, for the *K_JIC_* -evaluation (*B* > 10 *J_Q_*/*σ_Y_* and *b*_0_ > 10 *J_Q_*/*σ_Y_*).

## 3. Results

**Figure 2:**
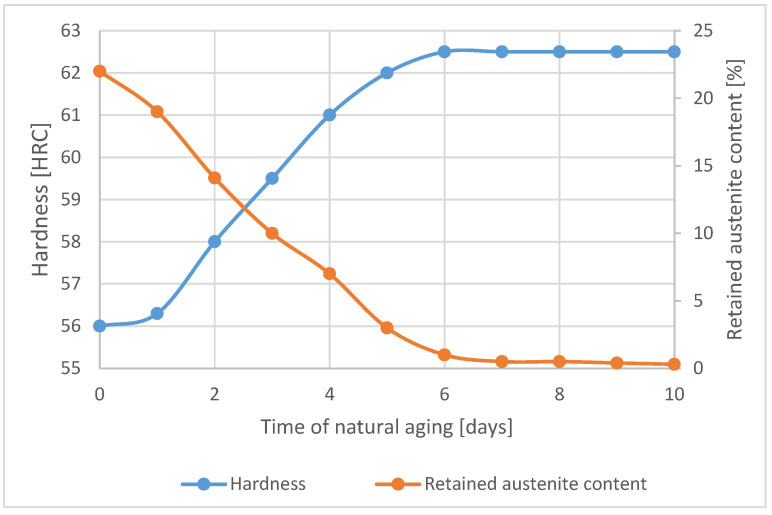
Hardness increase of experimental KAB steel A, during natural aging after quenching in oil from 900 °C.

The current steel was designed in order to exploit the possibilities of room temperature formation of bainite better. The KAB steel A was alloyed with a lower content of austenite stabilizing elements, but also developed a fine grain size due to pronounced micro-alloying with Ti and Nb, resulting in initially high content of retained austenite of 22%. The hardness increased during room temperature aging, and the retained austenite content was monitored, with the results summarized in Figure 2. After the hardness stabilized after about 6 days, the retained austenite was determined at a value of only 0.3%, whereby the reaction can be considered complete.

**Figure 3:**
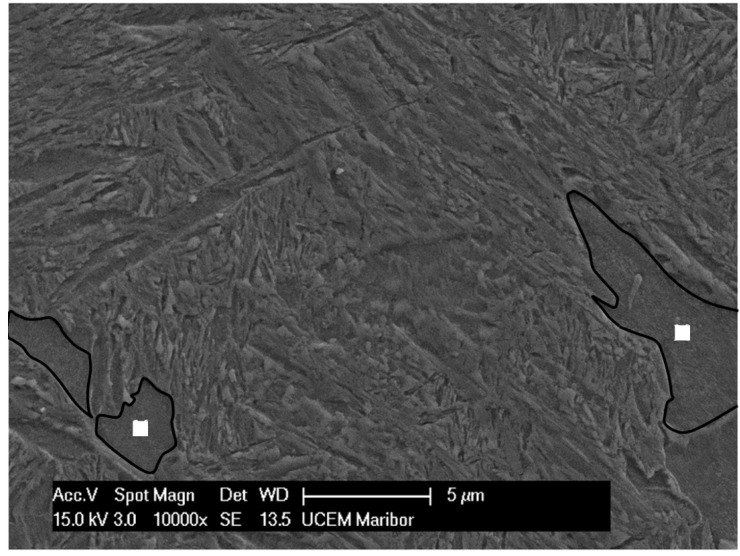
FESEM image of fine bainitic microstructure of the experimental KAB steel.

**Figure 4:**
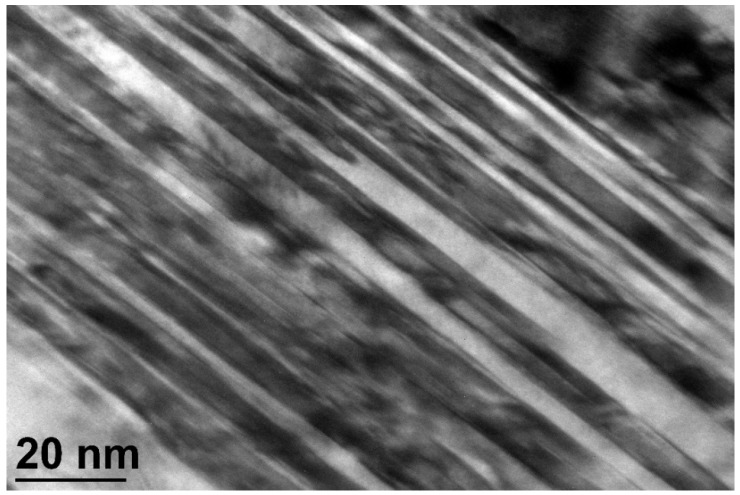
HRTEM image of a fine bainitic microstructure of KAB steel showing individual bainitic ferrite plates. When observed in bright field mode, the bainitic ferrite plates (α) appeared light, and the retained austenite (γ) dark.

The regions where the bainite lath structure is notably finer correspond to the portion of retained austenite that transformed during natural aging and are marked with an square in Figure 3. The fine structure of the individual bainitic ferrite plates is shown in Figure 4.

**Figure 5:**
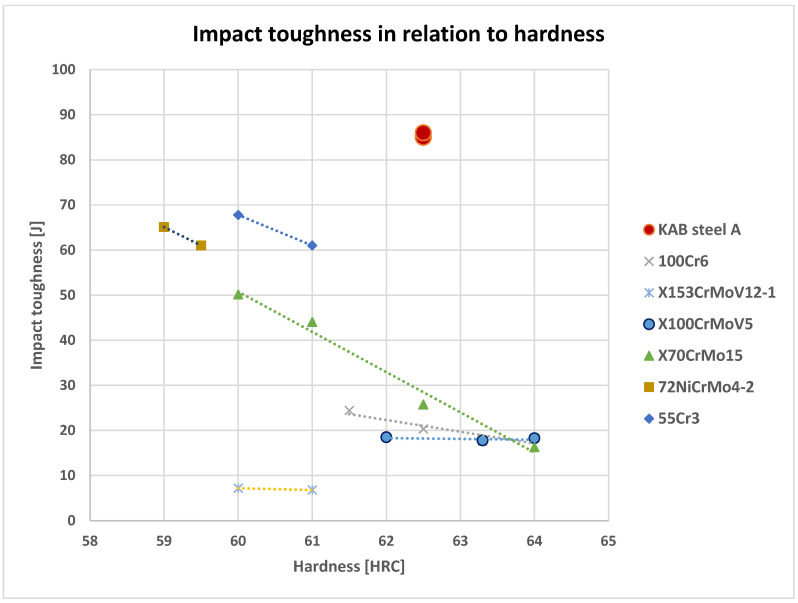
Toughness of experimental KAB steel compared to tool and low-alloy steels at similar hardness.

The fine microstructure with low retained austenite content resulted in the following mechanical properties summarized in Table 2:

The impact toughness was much higher compared to previous results of nanostructured bainitic steels such as S250 [15], which does not exhibit natural aging behavior. Compared to conventional steels in this hardness range, such as 72NiCrMo4-2 and 100 Cr6 which have a fully martensitic microstructure and are tempered at similar temperatures, the toughness also significantly improved. The orientational values, commonly recorded when using such testing procedures, are summarized in Figure 5.

**Figure 6:**
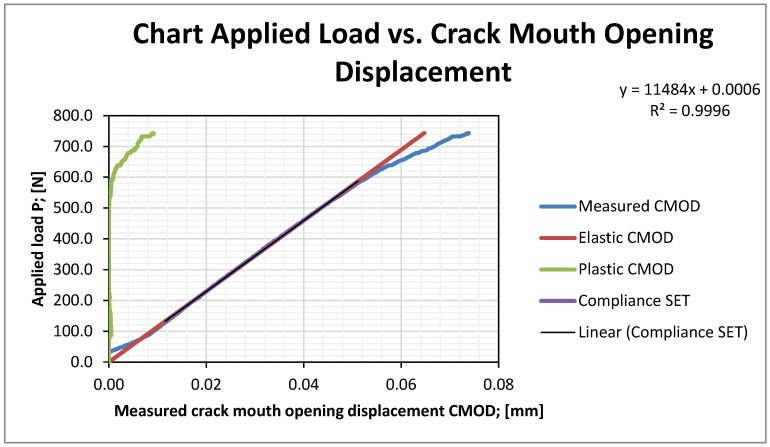
Force/CTOD (crack tip opening displacement) plot of test sample.

Crack mouth opening displacement (CMOD)-crack growth/force curves were generated for SENB specimens subjected to bending, as shown in Figure 6.

The different regions of crack initiation and propagation are clearly visible on the fracture surface. The boundary lines follow a slightly curved trajectory, but still meet the geometrical requirements described by the Standard. Single inclusions seem to be less important within the fatigue cracked region, where the crack propagates partially along the grain boundaries, preferably alongside larger grains.

**Figure 7:**
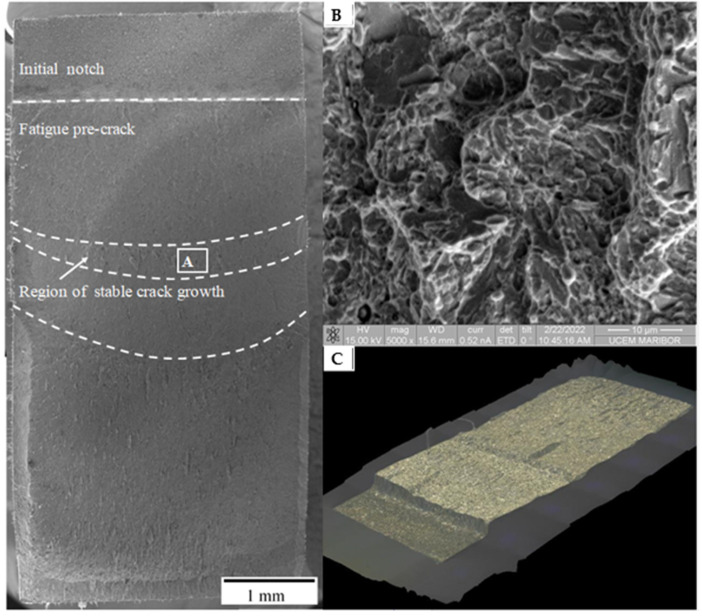
Fracture toughness specimen with fractographic detail: (**A**,**C**) Region of stable crack growth. (**B**) Isometric image of the fracture surface.

Fractography of the test sample has revealed a ductile fully transcrystalline character, as can be seen in Figure 7. The fracture surface of the crack initiation region (Figure 7A) is comparably coarser, whereas the crack propagation region is characterized by numerous fine dimples formed predominantly in the vicinity of non-metallic inclusions. The 3D shape of the physical crack is shown in Figure 7B.

**Figure 8:**
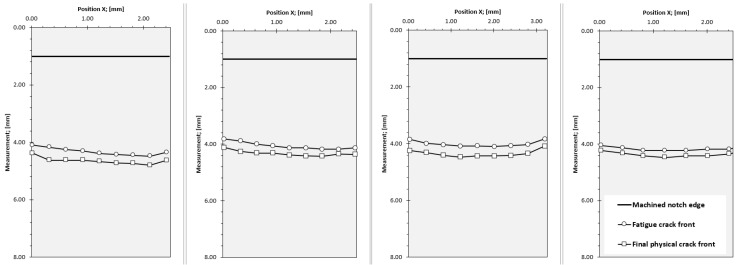
Fracture surface measurements of samples.

The crack measurements for the different samples are depicted in Figure 8.

**Figure 9:**
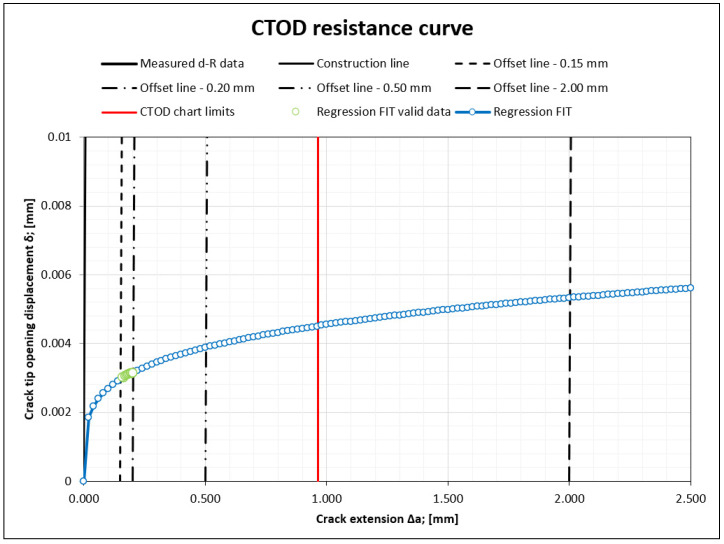
J integral resistance curve (sample 4).

From the measured data, J-integral resistance curves were constructed, as shown in Figure 9 for sample 4. In the construction of the chart crack, an extention of up to 1 mm was used in order to fullfil the test conditions according to the Standard.

**Figure 10:**
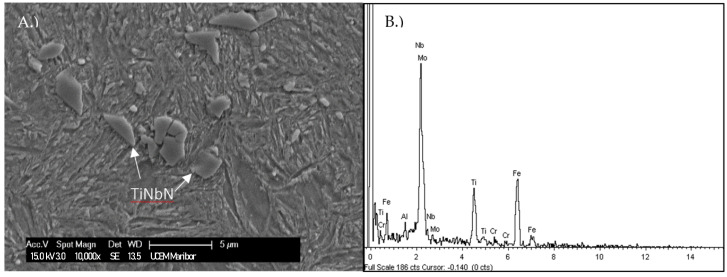
(**A**) Cluster of TiNbN inclusions adjacent to the fatigue crack, (**B**) corresponding EDX spectrum of the marked particles.

It is assumed that the observed non-metallic inclusions visible on the fracture surface correspond to clusters of TiNbN inclusions, as shown in Figure 10. These are thought to provide easy paths for crack propagation, and result in the apparently coarse fracture surface.

## 4. Discussion

In principle, there is no lower temperature limit for the formation of bainite. In this steel, bainite formed at room temperature during natural aging. The austenite transformed into bainite, as this is energetically more favorable due to the lower strain energy produced by very fine plates of bainitic ferrite as opposed to martensite. The prior austenite grain size has a very pronounced effect on martensite formation, but a negligible effect on bainite formation. Due to the very fine scale of the retained austenite, additional strain energy is required for martensite formation, effectively suppressing the Ms temperature below room temperature [15]. The very fine plates of bainitic ferrite produce very low strain, which is proportional to the size of the plate [16].

The impact toughness of nanostructured bainitic steels increased as the retained austenite fraction reduced to very low levels. This is consistent with our previous theoretical discussions [13]. It has also been reported that the retained austenite in such microstructures is transformed into martensite during impact testing [17]. Martensite is formed in front of the crack tip [18] and has high carbon content, which is known to be very brittle and promote cracking [19]. This is not observed during fatigue crack growth, and such steels exhibit relatively high fracture toughness, which is in large part attributed to the retained austenite content. Since the retained austenite content of KAB steel is negligible for comparable values of fracture toughness, the current results suggest that resistance to crack growth is predominantly a consequence of the fine bainitic microstructure.

The crack front shape in the broken base metal SENB specimens showed that blunting occurred along the crack, such that it was curved more deeply in the center than the free edges of the cross-section (Figure 7). This is because the specimen affirms to plane-strain constraint conditions in the middle thickness and is less constrained towards the outer surfaces.

**Figure 11:**
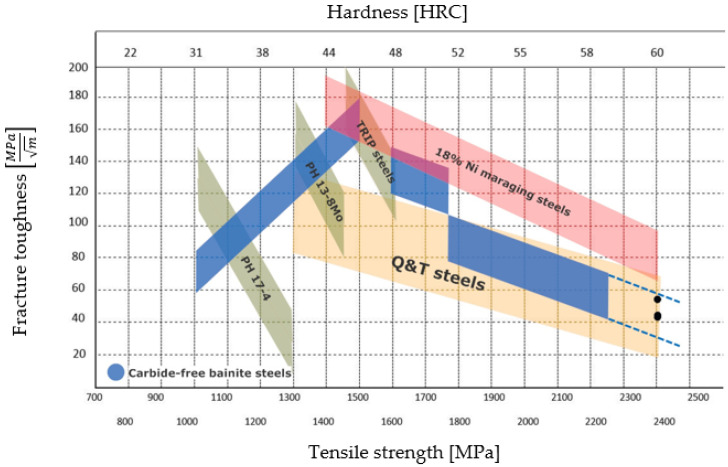
Fracture toughness data for some ultra-high strength steels.

To put the current results into perspective, some data for fracture toughness K_IC_ values in relation to Rockwell C hardness were plotted in Figure 11. The data were adapted from [20] and the available literature [21,22,23].

The Standard requirement of stable crack growth was met; however, the geometric conditions were not satisfied. Therefore, it was not possible to obtain true values of K_IC_, and the current results only yielded K_Q,_ which is provisional K_IC_ fracture toughness. K_Q_ represents the conditional fracture toughness at a critical point [24]. It can be seen that the obtained values are in accordance with the trend, as illustrated by the extrapolated lines in Figure 10. They also correlate well with the steel’s hardness, despite the low retained austenite content. The obtained K_Q_ values and true K_IC_ values of the 4 samples are summarized in Table 3 and have been marked on the graph in Figure 10.

## 5. Conclusions

It can be seen that, through the development of a very fine carbide-free bainitic microstructure, very high strength levels can be obtained within steels of lean composition. The heat treatment times are currently very long, but only present a marginal increase in cost, as the treatment is performed at room temperature. It is assumed that the required times for natural aging can be shortened significantly without a decrease in hardness by using alloys with lower contents of alloying elements, which decrease the driving force for ferrite formation. The strength of the current steel originates mainly from the very fine structure and dislocation strengthening mechanisms, whereby the role of carbon needs to be elucidated further. It is, however, reasonable to assume a further increase in strength by increasing the volume fraction of the room-temperature bainite. It is shown that the concept of applying natural aging in order to remove essentially all remnants of retained austenite is beneficial, simultaneously increasing the hardness and impact toughness of the steel, especially as the latter improved significantly compared to conventional steels at a similar hardness.

The current study has not shown any detrimental effect of a low retained austenite content on the fracture toughness, as the microstructure provides adequate blunting at the crack tip. The comparably coarser fracture surface in the crack initiation region is thought to be connected to clusters of TiNbN inclusions. The overall fracture toughness of the current steel is estimated to be comparable to Q&T steels of similar strength and comparable to the extrapolated value for these groups of steels.

The observed toughness is thought to be correlated with the theoretically immense dislocation density, whereby the majority are introduced by the formation of bainite subunits. Such dislocation is considered to be significantly more mobile compared to that created by processes such as severe plastic deformation, thereby further outlining that phase transformations offer several advantages and therefore present the preferred route for obtaining nanostructured steels. Nano-structuring using phase transformations also presents the possibility of large-scale production, whereby the scaling of the newly developed steel grade to industrial charges is currently the main goal.

This will also provide larger samples of higher purity to evaluate the fracture toughness more accurately. Further work should also be directed toward the characterization of complex mechanical properties, such as the high strain rate behavior of the newly developed steel. Now that a K_Q_ value has been found, a series of validation requirements are needed in future work to verify that the test results meet all the constraints and that the K value found is the critical K_IC_.

## 6. Patents

This work relates to the original patent for kinetically activated bainite steels (KAB), number SI 25891 A.

## Figures and Tables

**Table 1:** Nominal compositions of characterized steels (in wt %).

	C	Si	Mn	Mo	Cr	V	Al	Ti	Nb	Other	Fe
KAB Steel	0.6	1.2	1.5	0.6	1.5	0.22	1.5	0.015	0.02	<1	Bal.

**Table 2:** Range of work hardness and corresponding strength and toughness of the newly developed steel.

Hardness [HRC]	62.5
Yield strength (eng.) [MPa]	1950
Tensile strength (eng.) [MPa]	2397
Reduction in area [%]	9.6
Elongation to fracture [%]	8
Impact toughness [J]	85

**Table 3:** Summary of results.

	Sample 1	Sample 2	Sample 3	Sample 4
Evaluated SIF from JQ KJ_IC_; [MPa∙m^1/2^]	54.7	46.8	44.4	54.1

## Data Availability

All data presented in this paper is available upon request to one of the authors.

## References

[1] Antolovich S.D., Chanani G.R. (1972). Subcritical crack growth of trip steels in air under static loads. Eng. Fract. Mech..

[2] Antolovich S.D., Singh B. (1971). On the toughness increment associated with the austenite to martensite phase transformation in TRIP steels. Metall. Mater. Trans. B.

[3] Antolovich S.D., Fahr D. (1972). An experimental investigation of the fracture characteristics of trip alloys. Eng. Fract. Mech..

[4] Lacroix G., Pardoen T., Jacques P.J. (2008). The fracture toughness of TRIP-assisted multiphase steels. Acta Mater..

[5] Soliman M., Palkowski H. (2007). Ultra-fine Bainite Structure in Hypo-eutectoid Steels. ISIJ Int..

[6] Chatterjee S., Bhadeshia H.K.D.H. (2007). Transformation induced plasticity assisted steels: Stress or strain affected martensitic transformation?. Mater. Sci. Technol..

[7] Olson G.B., Cohen M. (1982). Stress-Assisted Isothermal Martensitic Transformation Application to TRIP Steels. Metall. Trans. A.

[8] Babu S.S., Vogel S., Garcia-Mateo C., Clausen B., Morales-Rivas L., Caballero F.G. (2013). Microstructure evolution during tensile deformation of a nanostructured bainitic steel. Scr. Mater..

[9] Garcia-Mateo C., Caballero F.G. (2005). Ultra-high-strength Bainitic Steels. ISIJ Int..

[10] Bhadeshia H.K.D.H. (2010). Nanostructured bainite. Proc. R. Soc. A Math. Phys. Eng. Sci..

[11] Al Hamdany A., Al Fattal D., Jabbar T.A., Bhadeshia H.K.D.H. (2012). Estimation of fracture toughness of tempered nanostructured bainite. Mater. Sci. Technol..

[12] García-Mateo C., Caballero F.G. (2005). The Role of Retained Austenite on Tensile Properties of Steels with Bainitic Microstructures. Mater. Trans..

[13] Kirbiš P., Pirtovšek T.V., Anžel I., Bruncko M. Designing tough nanostructured bainite. Proceedings of the Materials Science and Technology Conference and Exhibition 2017.

[14] ASTM International (1997). Standard Test Method for Plane-Strain Fracture Toughness of Metallic Materials 1.

[15] Takaki S., Fukunaga K., Syarif J., Tsuchiyama T. (2004). Effect of Grain Refinement on Thermal Stability of Metastable Austenitic Steel. Mater. Trans..

[16] Christian J.W., Olson G.B., Cohen M. (1995). Classification of displacive transformations: What is a martensitic transformation?. J. Phys. IV Proc..

[17] Fielding L.C.D., Jones N.G., Walsh J., Van Boxel S., Blackmur M.S., Lee P.D., Withers P.J., Stone H.J., Bhadeshia H.K.D.H. (2016). Synchrotron analysis of toughness anomalies in nanostructured bainite. Acta Mater..

[18] Tsai Y.T., Chang H.T., Huang B.M., Huang C.Y., Yang J.R. (2015). Microstructural characterization of Charpy-impact-tested nanostructured bainite. Mater. Charact..

[19] Chatterjee S., Bhadeshia H.K.D.H. (2006). TRIP-assisted steels: Cracking of high-carbon martensite. Mater. Sci. Technol..

[20] Olson G.B. (2001). Systems design of high performance stainless steels I. Conceptual and computational design. J. Comput. Mater. Des..

[21] Igwemezie V.C., Agu P.C. (2014). Development of Bainitic Steels for Engineering Applications. Int. J. Eng. Res. Technol..

[22] Bhadeshia H.K.D.H. (2002). The Design of Strong Tough and Affordable Engineering Alloys: 37th John Player Memorial Lecture.

[23] Okorafor O.E. (2014). Fracture toughness of M2 and H13 alloy tool steels. Mater. Sci. Technol..

[24] Zhu X.K., Joyce J.A. (2012). Review of fracture toughness (G, K, J, CTOD, CTOA) testing and standardization. Eng. Fract. Mech..

